# PICAML-MLLT10融合基因阳性急性髓系白血病4例报告并文献复习

**DOI:** 10.3760/cma.j.issn.0253-2727.2023.08.014

**Published:** 2023-08

**Authors:** 德媛 胡, 凯 沈, 虞莎 郭, 苏宁 陈

**Affiliations:** 苏州大学附属第一医院，江苏省血液研究所，国家血液系统疾病临床研究中心，国家卫生健康委员会血栓与止血重点实验室，苏州 215006 Jiangsu Institute of Hematology, National Clinical Research Center for Hematologic Diseases, NHC Key Laboratory of Thrombosis and Hemostasis, The First Affiliated Hospital of Soochow University, Suzhou 215006, China

10号染色体上的PICALM基因与11号染色体上MLLT10基因融合形成PICALM-MLLT10融合基因，是淋巴细胞系及髓系血液系统恶性肿瘤中较为罕见的融合基因，该融合基因参与诱导白血病的发生且与不良预后密切相关[1]–[3]。由于发生率低，既往报道少，异基因造血干细胞移植（allo-HSCT）对于该类患者的疗效更是未知。我们回顾性分析了2017年至2021年在苏州大学附属第一医院接受allo-HSCT治疗的4例PICALM-MLLT10融合基因阳性急性髓系白血病（AML）患者的临床和实验室资料并进行相关文献复习。

## 病例资料

例1，男，28岁，主诉咳嗽伴发热2 d至当地医院就诊。入院血常规：WBC 16.0×10^9^/L，HGB 86 g/L，PLT 122×10^9^/L；骨髓象：急性髓系白血病，不除外AML-M5（原幼单核细胞占85.5％，可见Auer小体）；白血病免疫分型：异常髓系原始细胞占有核细胞90％，符合AML表型。骨髓活检：骨髓增生大致正常（50％～60％），幼稚阶段细胞弥漫增生，少量偏成熟阶段粒红系细胞散在分布，巨核细胞不少，分叶核为主，网状纤维染色（MF-0级）。染色体核型分析：所分析20个细胞中有16个为+4，t（10;11）（p13;q21），其中一个伴有+21，可见克隆性异常+4，t（10;11）；43种急性白血病相关融合基因检测阴性。RNA测序检出PICALM-MLLT10融合基因，二代测序检出ASXL1-p.Gly646fs（变异比例30.53％）、PHF6-p.Ala41fs（变异比例72.1％）。予IAC方案诱导治疗后达完全缓解（CR），同日行腰穿鞘注，脑脊液未见异常。以地西他滨联合高剂量阿糖胞苷巩固1个疗程。期间复查骨髓均处于CR状态，行无关供者全相合造血干细胞移植，移植过程顺利，目前处于CR状态。

例2，男，35岁，因“发热3 d”入院。入院血常规：WBC 11.27×10^9^/L，HGB 133 g/L，PLT 177×10^9^/L；骨髓象：骨髓增生明显活跃，原幼细胞占83％，红系缺如，成熟淋巴细胞占13％，全片未见巨核细胞；白细胞免疫分型：分析33.6％幼稚细胞群体，各系分化不明，符合急性未分化型白血病（AUL）。染色体核型分析：正常核型。43种急性白血病相关融合基因检测阴性。确诊为AUL。予地西他滨预处理后，予VP+IAG方案联合化疗，骨髓未达到CR。骨髓活检示：髂后增生性骨髓，见弥漫分布的幼稚细胞，免疫组化失表达。二代测序检出BRAF-P523L（变异比例4.7％）、CSMD1-A534T（变异比例4.7％）、PAX5-V28M（变异比例3.8％）。RNA测序检出PICALM-MLLT10融合基因。予高剂量阿糖胞苷+米托蒽醌方案再诱导化疗后骨髓达部分缓解（PR），患者初诊时骨髓标本FISH检测到CSF1R微缺失及PDGFRB重排，末次化疗后加用达沙替尼治疗。期间腰穿鞘注脑脊液结果未见异常。患者在PR状态下行挽救性移植，移植后8个月复发，复发后2个月病情恶化死亡。

例3，男，26岁，主诉咳嗽咳痰伴胸闷气促3 d至当地医院就诊，入院血常规：WBC 52.19×10^9^/L，HGB 142 g/L，PLT 118×10^9^/L；骨髓象：原始单核细胞48.5％，幼稚单核细胞10％；白血病免疫分析：分析85.2％的幼稚细胞群体，为髓系抗原表达。43种急性白血病相关融合基因阴性。染色体核型46, XY, del（1）（q11）, add（9）（p24）, der（9）t（1,9）（q21;q34）, t（10;11）（p13;q21）［cp12］/46,XY[8]，予IA方案诱导达CR，以IA方案巩固，行腰穿提示患者合并中枢神经系统白血病（CNSL），予规律鞘内注射治疗直至脑脊液复查正常。予中剂量阿糖胞苷第二次巩固。患者骨髓缓解3个月后首次复发，二代测序检出NOTCH1-p.Glu2534fs（变异比例21.38％）、TP53-p.Asn239Asp（变异比例21％）、TP53-p.Gly245Ser（变异比例3％）、PHF6-p.Arg274*（变异比例50％）、SUZ12-p.Thr596fs（变异比例27.67％）。再次予以HA方案联合替莫唑胺诱导未达到CR，阿扎胞苷再诱导后获得CR2，RNA测序检测提示PICALM-MLLT10融合基因转录本，行allo-HSCT。最终患者因复发死亡。

例4，女，27岁，主诉颈部左侧痛性淋巴结肿大而就诊，入院血常规：WBC 39.42×10^9^/L，HGB 126 g/L，PLT 198×10^9^/L；骨髓象：原始细胞占84％。白细胞免疫分型：分析95％的幼稚细胞群体，为髓系抗原表达，符合AML伴CD7表达。融合基因检测：PICALM-MLLT10融合基因阳性。染色体核型46,XX,? add（10）（p11）del（11）（q21）, add（19）（q13）, inc[12]/46,XX[4]；二代测序检出TET2错义突变（变异比例45.82％）、PHF6无义突变（变异比例44.99％）、NRAS错义突变（变异比例46.34％）。予以AZA+HDA方案诱导，因消化道反应停止化疗，改用FLAG方案+维奈克拉治疗后达CR，继以FLAG+维奈克拉、维奈克拉+半程IAG巩固2个疗程，患者在CR1状态下行allo-HSCT，目前仍在缓解中。

## 讨论及文献复习

CALM也称为PICALM基因，位于染色体11 q23，其编码蛋白是介导细胞内吞作用的接合蛋白，定位于胞质，通过出核基序能与胞膜上的网格蛋白结合[4]。AF10也称MLLT10基因，位于染色体 10p12，其编码的蛋白质是克隆MLL染色体易位的配偶体[5]。PICALM-MLLT10融合基因是淋巴细胞系及髓系血液系统恶性肿瘤中较为罕见的融合基因，是染色体易位导致10号染色体PICALM基因与11号染色体MLLT10基因融合而成。

CALM-AF10最初发现于弥漫性组织细胞性淋巴瘤患者淋巴肿瘤组织内，并由此克隆建立U937细胞系[6]。PICALM-MLLT10融合基因是急性T淋巴细胞白血病（T-ALL）患者最常见的融合基因，占T-ALL患者的7％～10％[1]，占AML患者的0.3％～2％[7]。患者临床表现为肝脾肿大、纵隔肿块、CNSL等，对常规化疗药物不敏感且易早期复发，预后不佳[1],[7]–[9]。由于发病率较低，目前大宗报道较少。Borel等[10]在2010年报道了1例AML-M0伴t（10;11）的患者在最初诊断后存活了15年，患者进行allo-HSCT并在11年后复发，在复发阶段检出了PICALM-MLLT10融合基因，后进行了二次allo-HSCT。结合患者初诊时有10和11号染色体易位，考虑患者在初诊即存在该融合基因。例3在初诊时染色体核型分析示10和11号染色体易位但未行RNA测序，复发后检出PICALM-MLLT10融合基因。可见，RNA测序筛查是复发/难治AML患者融合基因检测的有效手段。但结合既往文献研究，我们暂不考虑AML复发后继发PICALM-MLLT10融合基因的可能性，复发后检出该融合基因可能是患者初诊时由于检测技术的限制未检出。Borel等[7]在2012年报道了18例伴PICALM-MLLT10融合基因阳性的AML患者，是迄今为止最大宗的报道，18例融合基因阳性AML患者在总生存期、2年无病生存率以及2年总生存率方面与融合基因阴性患者无明显差异。其中7例患者接受了造血干细胞移植，3例患者移植后发。Huh等[11]报道了2例移植患者，1例患者在移植后4个月后复发，另1例患者移植后1个月随访期内处于CR状态。我们报道的4例患者中，在CR2状态及NR状态下移植的患者最终都复发死亡，2例患者在CR1状态下移植后分别缓解6个月及20个月，目前仍在缓解中。国内报道的PICALM-MLLT10融合基因阳性的AML患者仅2例，1例患者确诊2个月后死亡，另一例患者多次化疗仍未缓解，确诊11个月后死于脏器衰竭，均未行allo-HSCT，提示此类患者疾病进展快，预后不佳[12]。以往报道显示PICALM-MLLT10融合基因阳性的AML患者移植后复发风险仍旧很高，但移植似乎可以延长患者的生存期，这有待更大的样本去验证。

在PICALM-MLLT10融合基因阳性白血病中进行的研究中，常发现HOX基因家族表达上调，此被认为干扰了正常的造血分化，从而导致白血病的发生[13]–[14]。从治疗角度而言，目前尚无针对PICALM-MLLT10融合基因阳性AML的靶向药物和统一的治疗方案，此类患者单纯化疗预后不佳，移植可以延长患者的生存，但此类患者并非均有机会接受移植，寻找靶向药物至关重要。最近研究发现CALM-AF10蛋白依赖于Jak1激活JAK/STAT炎症信号通路[15]，且Jak1抑制剂在AF10重排的AML模型中显示出强大的抗癌活性。先前研究表明，组蛋白甲基转移酶DOT1L是AF10重排AML中一个有效的靶点[16]，但小分子DOT1L抑制剂pinometostat对AML的疗效有限。而Conway等[13],[17]研究发现核输出蛋白XPO1对于Hoxa基因上调是必不可少的，而且XPO1抑制剂对于AF10重排AML患者疗效较好。因此，XPO1抑制剂Selinexor有望成为治疗PICALM-MLLT10阳性AML的新型靶向药物。

随着RNA测序技术的普及，PICALM-MLLT10融合基因等少见分子学异常的检出率会逐渐升高，因此对此类病例临床特征及治疗转归的总结是必要的。t（10;11）（p13;q21）重排作为存在于多种血液恶性肿瘤中的复现性细胞遗传学异常，RAN测序是其有效的检测手段。

## References

[1] Asnafi V, Radford-Weiss I, Dastugue N (2003). CALM-AF10 is a common fusion transcript in T-ALL and is specific to the TCRgammadelta lineage[J]. Blood.

[2] van Grotel M, Meijerink JP, Beverloo HB (2006). The outcome of molecular-cytogenetic subgroups in pediatric T-cell acute lymphoblastic leukemia: a retrospective study of patients treated according to DCOG or COALL protocols[J]. Haematologica.

[3] Dreyling MH, Schrader K, Fonatsch C (1998). MLL and CALM are fused to AF10 in morphologically distinct subsets of acute leukemia with translocation t(10;11): both rearrangements are associated with a poor prognosis[J]. Blood.

[4] Scotland PB, Heath JL, Conway AE (2012). The PICALM protein plays a key role in iron homeostasis and cell proliferation[J]. PLoS One.

[5] Chaplin T, Bernard O, Beverloo HB (1995). The t(10;11) translocation in acute myeloid leukemia (M5) consistently fuses the leucine zipper motif of AF10 onto the HRX gene[J]. Blood.

[6] Sundström C, Nilsson K (1976). Establishment and characterization of a human histiocytic lymphoma cell line (U-937)[J]. Int J Cancer.

[7] Borel C, Dastugue N, Cances-Lauwers V (2012). PICALM-MLLT10 acute myeloid leukemia: a French cohort of 18 patients[J]. Leuk Res.

[8] Lo Nigro L, Mirabile E, Tumino M (2013). Detection of PICALM-MLLT10 (CALM-AF10) and outcome in children with T-lineage acute lymphoblastic leukemia[J]. Leukemia.

[9] DiNardo CD, Tang G, Pemmaraju N (2015). Acute myeloid leukemia with t(10;11): a pathological entity with distinct clinical presentation[J]. Clin Lymphoma Myeloma Leuk.

[10] Borel C, Huynh A, Chaufour X (2010). Uterine chloroma, aortic thrombus and CALM/AF10 acute myeloid leukemia[J]. Leuk Res.

[11] Huh JY, Chung S, Oh D (2010). Clathrin assembly lymphoid myeloid leukemia-AF10-positive acute leukemias: a report of 2 cases with a review of the literature[J]. Korean J Lab Med.

[12] 鲁 淑婷, 姚 金红, 李 少阳 (2021). 伴PICALM-MLLT10的急性非淋巴细胞白血病2例报告并文献复习[J]. 重庆医学.

[13] Conway AE, Haldeman JM, Wechsler DS (2015). A critical role for CRM1 in regulating HOXA gene transcription in CALM-AF10 leukemias[J]. Leukemia.

[14] Caudell D, Zhang Z, Chung YJ (2007). Expression of a CALM-AF10 fusion gene leads to Hoxa cluster overexpression and acute leukemia in transgenic mice[J]. Cancer Res.

[15] Chen BR, Deshpande A, Barbosa K (2021). A JAK/STAT-mediated inflammatory signaling cascade drives oncogenesis in AF10-rearranged AML[J]. Blood.

[16] Stein EM, Garcia-Manero G, Rizzieri DA (2018). The DOT1L inhibitor pinometostat reduces H3K79 methylation and has modest clinical activity in adult acute leukemia[J]. Blood.

[17] Conway AE, Scotland PB, Lavau CP (2013). A CALM-derived nuclear export signal is essential for CALM-AF10-mediated leukemogenesis[J]. Blood.

